# Left-sided colorectal cancer distinct in indigenous African patients compared to other ethnic groups in South Africa

**DOI:** 10.1186/s12885-022-10185-3

**Published:** 2022-10-24

**Authors:** Michelle McCabe, Clement Penny, Pumza Magangane, Sheefa Mirza, Yvonne Perner

**Affiliations:** 1 Division of Anatomical Pathology, School of Pathology, Faculty of Health Sciences, University of the Witwatersrand, National Health Laboratory Services, Johannesburg, 2193 South Africa; 2 Division of Human Genetics, School of Pathology, Faculty of Health Sciences, University of the Witwatersrand, National Health Laboratory Services, Braamfontein, Johannesburg, 2000 South Africa; 3 Department of Internal Medicine, Faculty of Health Sciences, University of the Witwatersrand, Parktown, Johannesburg, 2193 South Africa

**Keywords:** Colorectal cancer, African, Microsatellite stable, Low level microsatellite instability, BAT25, BAT26

## Abstract

**Introduction:**

A large proportion of indigenous African (IA) colorectal cancer (CRC) patients in South Africa are young (< 50 years), with no unique histopathological or molecular characteristics. Anatomical site as well as microsatellite instability (MSI) status have shown to be associated with different clinicopathological and molecular features. This study aimed to ascertain key histopathological features in microsatellite stable (MSS) and low-frequency MSI (MSI-L) patients, to provide insight into the mechanism of the disease.

**Methods:**

A retrospective cohort (2011–2015) of MSS/MSI-L CRC patient samples diagnosed at Charlotte Maxeke Johannesburg Academic Hospital was analyzed. Samples were categorized by site [right colon cancer (RCC) versus left (LCC)], ethnicity [IA versus other ethnic groups (OEG)] and MSI status (MSI-L vs MSS). T-test, Fischer’s exact and Chi-square tests were conducted.

**Results:**

IA patients with LCC demonstrated an increased prevalence in males, sigmoid colon, signet-ring-cell morphology, MSI-L with BAT25/26 marker instability and advanced disease association.

**Conclusion:**

This study revealed distinct histopathological features for LCC, and suggests BAT25 and BAT26 as negative prognostic markers in African CRC patients. Larger confirmatory studies are recommended.

## Introduction

Right-sided colon cancer (RCC) and left-sided colon cancer (LCC) show distinct mechanisms of development and are associated with different clinicopathological features [1–3]. During embryological development, the RC develops from the midgut and the LC from the hindgut, supporting the view that RCC and LCC develop through different developmental/embryological pathways genetic mechanisms [1, 4]. The incidence of RCC (~ 30%) is lower than LCC (~ 70%). RCC presents with larger tumours, a higher rate of tumour node metastases (TNM stage), mucinous features, and comprises of an overall poorer survival than LCC [1, 2, 4–6]. Older female patients are at higher risk of developing RCC compared to younger male patients associated with increased risk of developing LCC [1]. The literature shows population groups with a lower risk of developing CRC moving to high-risk areas acquire the risks associated with the new area, and this could be linked to dietary, environmental, cultural and genetic factors [1, 7]. This speaks to a possible role for the increasing incidence of CRC in indigenous African (IA) patients moving from rural to urbanized areas [8–10].

There are 3 main pathways involved in the development of CRC: 1) Microsatellite instability (MSI) caused by a defective mismatch repair (MMR) system, most often (~ 70–95%) caused by an alteration of MLH1,2) Chromosome instability (CIN) pathway which develops due to gross chromosomal changes and 3) the CpG island methylator phenotype (CIMP) pathway, arising through methylation of CpG islands in promoter sequences, leading to inactivation of tumour suppressor genes throughout the genome [11, 12]. MSI and CIMP tumours mostly occur in the right colon, whereas CIN CRC is associated with LCC [11, 13, 14]. Four main consensus molecular subtypes (CMS) were established in 2015. These are differentiated by unique molecular features: CMS1 (14%, MSI pathway, immune activation); CMS2 (37%, Canonical WNT/MYC pathway, epithelial signature); CMS3 (13%, epithelial and metabolic dysregulation), and CMS4 (23%; Mesenchymal TGF- β pathway; stromal invasions and angiogenesis) [11, 15].

To date, fewer research outputs on low frequency microsatellite instability (MSI-L) CRC have been published compared to MSI-H and MSS CRC. MSI-L is usually grouped with MSS CRC, as literature states all CRCs display some level of MSI [16, 17]. Some researchers interpret MSI-L tumours as precursors of MSI-H CRC, whereas others believe it to be a completely separate entity [18, 19]. MSI-L tumours have illustrated different clinicopathological features and have been considered to be a worse prognostic group in a few CRC studies [20–23]. Jass et al. reported that MSI-L LCC showed distinctive clinicopathological features, with a male predilection, a moderately differentiated histopathological grade, KRAS mutations, CIMP-Low status and DNA aneuploidy. In contrast, MSI-L RCC was found to occur more frequently in females, being associated with a serrated adenoma precursor lesion, mucinous adenocarcinoma histological subtype and poorly differentiated grade. BRAF mutations, CIMP-High status and diploid DNA content, features associated with a worse prognosis, were also associated with the MSI-L RCC group [24].

A disproportionate number of indigenous African (IA) patients display a younger age of onset (< 50 years of age) with no distinct histopathological features to assist with early diagnosis and management [25–28]. Previous work by McCabe et al. described MSI-H CRC in detail according to ethnicity groups and found an increased association of MSH2/MSH6 MMR protein expression loss in right sided CRC in young IA patients [29]. This study aims to characterize proficient MMR (MSS/MSI-L) CRC, by ethnicity (IA versus OEG) and anatomical site (LCC versus RCC), to potentially identify a unique subtype associated with young IA CRC patients.

## Methodology

### Patient demographics and tumour histopathological characterization

This retrospective study was conducted on a 5-year cohort (2011–2015) of 428CRC patient samples with known anatomical site and MMR status, diagnosed at the Charlotte Maxeke Johannesburg Academic Hospital (CMJAH) branch of the National Health Laboratory Service (NHLS). Informed consent was waived by the Human Research Ethics Committee (HREC) (Medical) of the University of the Witwatersrand for this study, as research was conducted under the institutional blanket ethics clearance (M10744) obtained from the HREC (Medical), which allows for research to be carried out on all archived pathology specimens without informed consent from study participants. Additional project-specific ethical clearance was also obtained from the HREC (Medical) (M120994), and all tests were performed according to the relevant guidelines and regulations. A total of 59 (14%) patient samples had a deficient MMR (dMMR) profile (MSI-H) and the remaining 369 (86%) a proficient MMR (pMMR) status. All pMMR CRC cases were categorized into 4 groups (see Table 1). Tumour site the main group: RCC (tumour primary site proximal to splenic flexure) versus LCC (tumour primary site distal from splenic flexure), further sub-grouped by ethnicity: Indigenous African (IA) versus Other Ethnic Groups (OEG) [Caucasian, Mixed Ancestry, Asian]. Demographic and histopathological information were analyzed within these categories i.e., gender, age, tumour subtype, grade, site, TNM stage (American Joint Committee on Cancer [AJCC] TNM stage), presence of tumour infiltrating lymphocytes (TIL) using the Klintrup-Mäkinen scoring assessment, Crohn’s-like inflammatory reaction (CLR), polyp subtype, venous, perineural and lymphatic invasion, all of which were obtained from histology reports. Immunophenotypic profiling of TIL was not conducted. No family histories were available from these reports.

**Table 1:** Descriptive analysis of MSS CRC cases diagnosed at CMJAH between (2011–2015). Categorized by site: LCC *vs* RCC and Ethnicity: Indigenous African (IA) *vs* Other Ethnic Group (OEG)

		**Total number cases (%)**	**Left-sided Colon Cancer (LCC)**		**Statistical analysis:**	**Right-sided Colon Cancer (RCC)**		**Statistical analysis:**
			**IA**	**OEG**		**IA**	**OEG**	
**Frequency**		**369**	**154 (42)**	**108(29)**		**63(17)**	**44(12)**	
**Demographical data**								
**GENDER**		**369**	**154**	**108**	*P* = 0.0111*	**63**	**44**	***P* = 0.6932**
**Male**		**209(57)**	98(64)*	**51(47)**		**34(54)**	**26(59)**	
**Female**		**160(43)**	**56(36)**	**57(53)**		**29(46)**	**18(41)**	
**AGE**		**365**	**152**	**107**	*P* < 0.0001***	**62**	**44**	*P* = 0.0103*
**Min–Max**		**15–92**	**20–90**	**28–92**		**15–79**	**25–86**	
**Mean ± SD**		**57 ± 14**	**53 ± 15**	**62 ± 13**		**54 ± 13**	**60 ± 11**	
**Median**		**59**	54***	62***		55*	61*	
**P25-P75 (Interquartile Range)**		**47–67**	**41–65**	**55–72**		**47–64**	**54–68**	
**95% CI**		**[55-58]**	**[51-56]**	**[60-65]**		**[51-57]**	**[57-65]**	
**Categorical Age**		**365**	**152**	**107**	*P* < 0.0001***	**62**	**44**	*P* < 0.0001***
** ≤ 50 years**		**107 (29)**	62(41)***	**15(14)**		24(39)***	**6(14)**	
** > 50 years**		**258(71)**	**90(59)**	**92(86)**		**38(61)**	**38(86)**	
***Histological characteristics***								
**TUMOUR SUBTYPE**		**360**	**152**	**104**	*P* = 0.0257*	**61**	**43**	***P* = 0.2608**
**Invasive Adenocarcinoma**		**329(91)**	**138(91)**	**100(96)**		**54(89)**	**35(81)**	
**Mucinous Adenocarcinoma**		**17(5)**	**4(3)**	**4(4)**		**3(5)**	**6(14)**	
**Signet Ring Cell Adenocarcinoma**		**14(4)**	**10(6)**	**0(0)**		**4(6)**	**2(5)**	
**TUMOUR SITE:**		**359**	**151**	**105**	*P* = 0.0221*	**61**	**42**	***P* = 0.2431**
**Left**	**Right**							
**Splenic Flexure**	**Hepatic Flexure**	**14(4)**	**5(3)**	**0(0)**		**6(10)**	**3(7)**	
**Descending colon**	**Ascending Colon**	**58(16)**	**21(14)**	**11(10)**		**11(18)**	**15(36)**	
**Sigmoid**	**Transverse Colon**	**83(23)**	46(31)*	**21(20)**		**10(16)**	**6(14)**	
**Rectum**	**Caecum**	**204(57)**	**79(52)**	**73(70)**		**34(56)**	**18(43)**	
**TUMOUR GRADE**		**348**	**148**	**95**	***P* = 0.0930**	**61**	**44**	***P* = 1.0000**
**Low Grade (LG)**		**316(91)**	**132(89)**	**91(96)**		**54(89)**	**39(89)**	
**High Grade (HG)**		**32(9)**	**16(11)**	**4(4)**		**7(11)**	**5(11)**	
**AJCC TNM STAGING**		**240**	**85**	**61**	***P* = 0.0922**	**57**	**37**	***P* = 0.4995**
**I-II**		**92(38)**	**31(38)**	**31(51)**		**20(36)**	**10(27)**	
**III-IV**		**148(62)**	**54(62)**	**30(49)**		**37(64)**	**27(73)**	
**TUMOUR INFILTRATING LYMPHOCYTES (TIL)**		**230**	**81**	**60**	***P* = 0.8512**	**54**	**35**	***P* = 0.6591**
**None**		**156(68)**	**58(72)**	**44(73)**		**34(63)**	**20(57)**	
**Mild-moderate**		**74(32)**	**23(28)**	**16(27)**		**20(37)**	**15(43)**	
**CROHN'S LIKE INFLAMMATORY RESPONSE**		**230**	**81**	**60**	***P* = 0.4406**	**54**	**35**	***P* = 0.3392**
**None**		**168(73)**	**62(77)**	**42(70)**		**41(76)**	**23(66)**	
**Mild-moderate**		**62(27)**	**19(23)**	**18(30)**		**13(24)**	**12(34)**	
**LYMPHATIC INVASION**		**285**	**115**	**76**	***P* = 0.2921**	**56**	**38**	***P* = 0.2896**
**Absent**		**203(71)**	**85(74)**	**62(82)**		**36(64)**	**20(53)**	
**Present**		**82(29)**	**30(26)**	**14(18)**		**20(36)**	**18(47)**	
**VENOUS INVASION**		**232**	**82**	**59**	***P* = 0.3937**	**55**	**36**	***P* = 1.0000**
**Absent**		**181(78)**	**68(83)**	**45(76)**		**41(75)**	**27(75)**	
**Present**		**51(22)**	**14(17)**	**14(24)**		**14(25)**	**9(25)**	
**PERINEURAL INVASION**		**241**	**91**	**60**	***P* = 0.1110**	**54**	**36**	***P* = 1.0000**
**Absent**		**187(78)**	**67(74)**	**51(85)**		**41(76)**	**28(78)**	
**Present**		**54(22)**	**24(26)**	**9(15)**		**13(24)**	**8(22)**	
**POLYPS**		**80**	**31**	**19**	***P* = 0.5516**	**16**	**14**	***P* = 0.7131**
**Tubular Adenoma (TA)**		**49(58)**	**21(68)**	**11(58)**		**10(63)**	**7(50)**	
**Tubulovillous Adenoma (TVA)**		**31(37)**	**10(32)**	**8(42)**		**6(37)**	**7(50)**	
**MSS/MSI-L PCR CONFIRMED CASES**		**233**	**94**	**85**	*P* = 0.0157*	**28**	**26**	***P* = 0.3582**
**MSS**		**189(81)**	**72(77)**	**77(91)**		**19(70)**	**21(81)**	
**MSI-L**		**44(19)**	**22(23)**	**8(9)**		**9(30)**	**5(19)**	
**MSI PCR MARKERS (MSI-L)**		**42**	**22**	**8**	*P* = 0.0138*	**7**	**5**	***P* = 0.2222**
**1 single unstable marker**					**BAT25/26**			**BAT25/26**
**BAT25**		**20(56)**	11(50)*	**5(62.5)**	**Vs**	**3((43)**	**1(20)**	***Vs***
**BAT26**		**15(22)**	11(50)*	**0(0)**	**NR21/24/27**	**3(43)**	**1(20)**	**NR21/24/27**
**NR21**		**4(12)**	**0(4)**	**2(25)**		**0(0)**	**2(40)**	
**NR24**		**3(10)**	**0(0)**	**1(12.5)**		**1(14)**	**1(20)**	
**NR27**		**0(0)**	**0(0)**	**0(0)**		**0(0)**	**0(0)**	

Levels of statistical significance indicated by an asterix *. *P*-values <0.05, is indicated with (*), *P*-values <0.01, (**), and *p*-values < than 0.001 (***)

### MSS versus MSI-L molecular subtyping

**Fig. 1:**
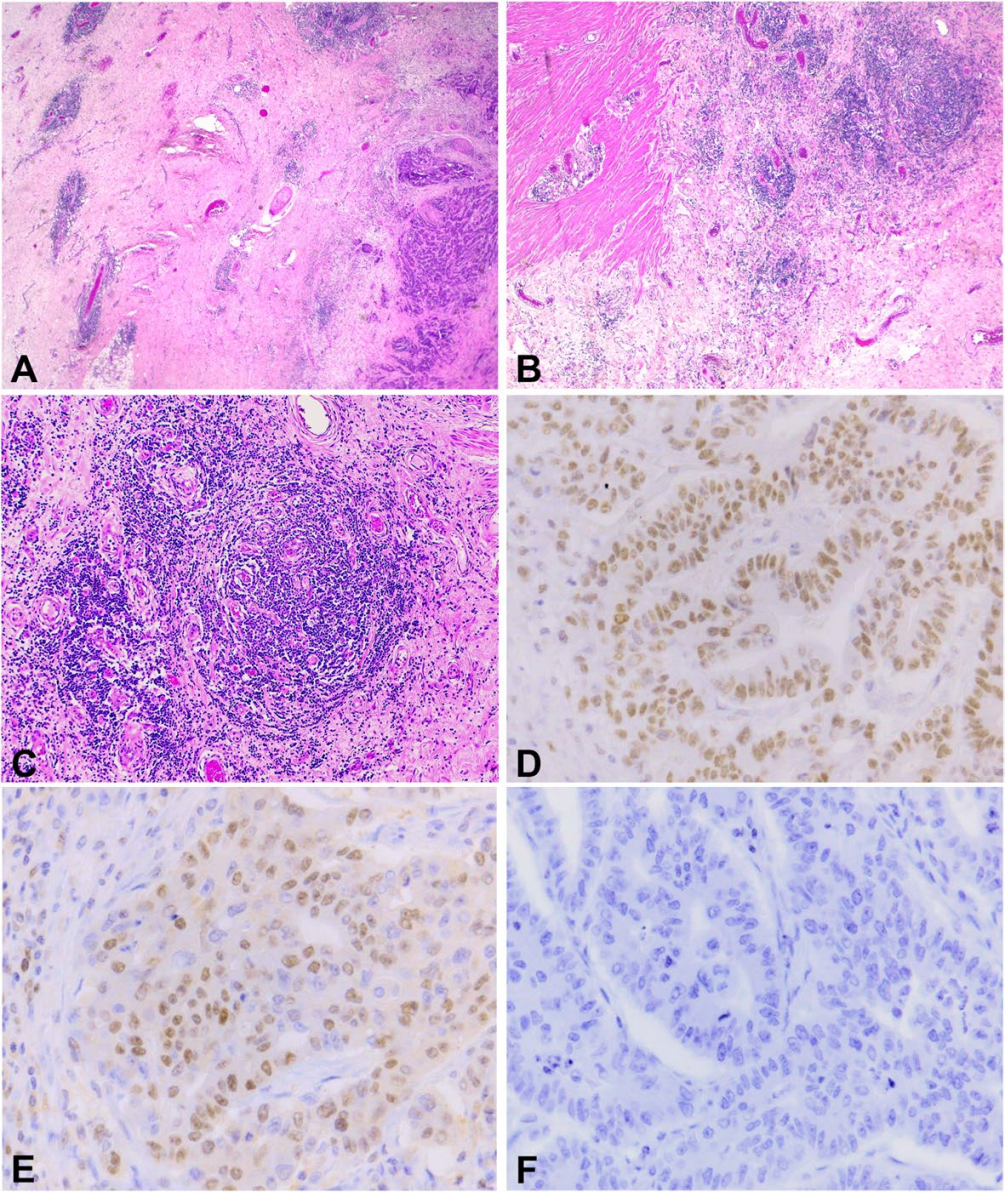
Haematoxylin and Eosin (**A-C**) images and Immunohistochemistry (**D-F**) images of colorectal (CRC) tissue sections. **A** Low power (40X) of serosa showing CRC and mild Crohn’s-like response (CLR). **B** Low (100X) power of serosa with moderate CLR. **C** Similar CLR at higher magnification (200X). **D** MSH2 retained nuclear expression (400X). **E** MLH6 retained nuclear expression (400X). **F** MLH1 loss of nuclear expression, showing MSIphenotype (400X)

**Fig. 2:**
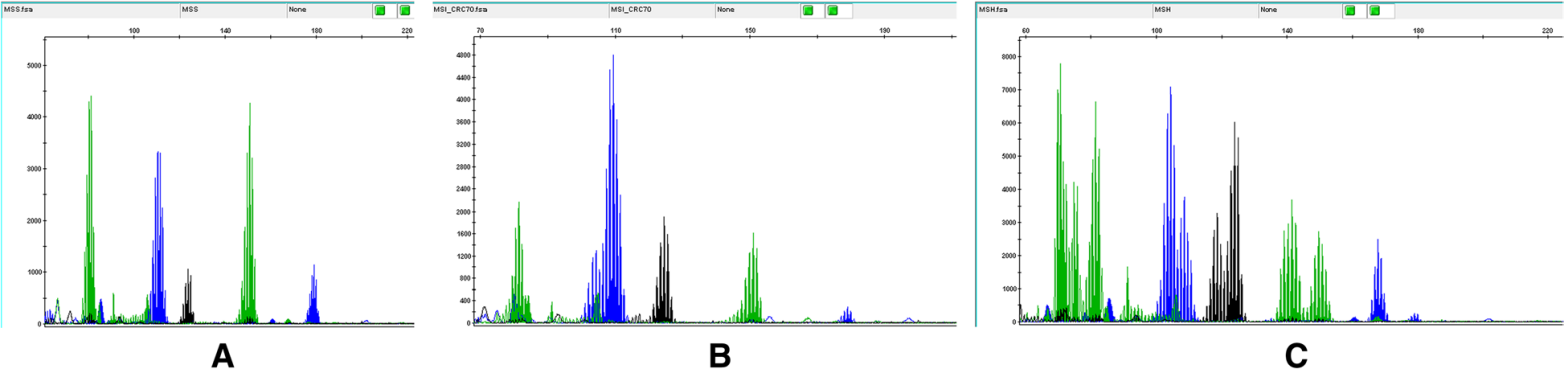
Electropherograms illustrating instability patterns using the 5-mononucleotide MSI PCR panel. **A** MSS: 5 markers stable **B** MSI-Low: 1 marker unstable **C** MSI-H: 5 markers unstable

Samples were screened for proficient MMR status through MMR immunohistochemistry (IHC) and/or MSI polymerase chain reaction (PCR). IHC images and MSI electropherograms are illustrated in Figs. 1 and 2 respectively. The MMR IHC panel included antibodies targeting MutL Homolog 1(MLH1), MutS Homolog 2 and 6 (MSH2/ MSH6) and Post Meiotic Segregation Homolog-2 (PMS2) protein expression. Only samples with a MMR proficient profile detected via IHC and a MSS or MSI-L profile determined via PCR were included in this cohort [29]. MSI PCR included the 5-mononucleotide PCR panel (NR27, NR21, NR24, BAT25 and BAT26); and a MSS or MSI-L result was ascribed when an allelic size varied in none or only one of the 5 markers respectively [29, 30]. The literature indicates that people of African descent exhibit normal variation within loci BAT25 and BAT26 [31–34]. A multipopulation study by Buhard et al. 2006, revealed approximately 10% show normal variation in one of five markers and 2% in 2 markers [33]. In silico analysis of BAT 25 and BAT 26 PCR primer sets were performed against the global 1000 Genomes dataset (www.internationalgenome.org/data) and local AWI-GEN dataset (https://www.wits.ac.za/research/sbimb/research/awi-gen). Data analysis from the 1000 Genomes dataset revealed 2 single nucleotide polymorphisms (SNPs) in each primer set and occurred at a rare frequency of 0.02%. AWI-GEN data analysis showed one SNP for BAT26 primers at position Chr2: 47,641,434, and occurred at a frequency of 0.5%. No SNPs were detected in BAT25 primer set. The 5-mononucleotide panel remained the assay of choice due to the additional 3 quasimonomorphic mononucleotide repeat markers found in Caucasian and African germ-line DNA, ensuring the panel is extremely sensitive in detecting somatic alterations in MSI-H tumours and distinguishing between MSS/MSI-L tumours. As recommended by the authors Suraweera et al. 2002, in tumour samples with instability in BAT25 and/or 26 markers with a proficient MMR profile via IHC, matched normal samples were assessed to establish the true instability status. In cases where these markers matched instability in normal colon tissue, the status was regarded as normal or germline variation and reported as MSS. In biopsies this was not possible due to the limited size and mixture of normal and neoplastic tissue, increasing the chance of contamination when assessing normal tissue.

### Statistical analysis

All data was collected in an excel sheet and statistical analysis was performed using Stata Intercooled 7.0 (Stata, College Station, TX, USA) and Graphpad Prism version 9.0 (Graphpad Software, La Jolla, CA, USA). Unpaired t-tests were used to assess differences between groups on continuous normally distributed variables while Fischer’s exact and Chi-square tests were used to assess associations between categorical variables; and a result with a *P* value less than 0.05 was considered statistically significant. Additionally, analysis of MSI status (MSI-L versus MSS and MSI-H CRC) stratified by tumour site (LCC versus RCC) was conducted to determine if any association occurred with demographic variables (gender, age and ethnic groups) as well as TNM stage, CLR and TIL (Table 2). R/Rstudio was used to perform multiple comparisons on MSI status stratified by tumour site with other categorical variables eg gender to ascertain pairwise associations and the False Discovery Rate (FDR) adjusted *p*-value (q-value) was reported and considered statistically significant if less than 0.05.

**Table 2:** Multiple comparison analysis of CRC cases diagnosed at CMJAH (2011–2015). Categorized by MSI status: MSI-L *vs* MSS *vs* MSI-H CRC and site: LCC *vs* RCC

	**Number of cases (%)**	**MSI-L CRC**		**MSS CRC**		**MSI-H CRC**		**Statistical analysis:** Multiple comparison analysis: ***(Associations between 2 groups italicized)***
	**CRC 2011–2015**	**A: LCC**	**B: RCC**	**C: LCC**	**D: RCC**	**E: LCC**	**F: RCC**	
**Frequency/ Prevalence**	**303**	**30(10)**	**12(4)**	**160(53)**	**42(14)**	**12(4)**	**47(15)**	
**GENDER**	303	30	12	160	42	12	47	X^2^ = 2.6877, df = 5
Male	160(53)	16(53)	6(50)	88(55)	24(57)	6(50)	20(43)	*P* = 0.748
Female	143(47)	14(47)	6(50)	72(45)	18(43)	6(50)	27(57)	*No association of sex*
**AGE**	303	29	12	160	42	12	47	FDR (q value = 0.8238) Q = 0.05
Min–Max	15–92	24–91	37–84	20–92	15–80	33–67	27–77	
Mean ± SD	57 ± 14	56 ± 17	59 ± 13	58 ± 15	58 ± 13	50 ± 12	53 ± 14	*E vs C: P* = 0.0415*
Median	59	56	60	59	58	52	51	*F vs C: P* = 0.0274*
P25-P75 (IQR)	48–69	40–72	47–67	48–69	53–67	38–61	43–62	
95% CI	[56-59]	[49-62]	[50–67]	[55-60]	[53-62]	[42-57]	[49-57]	
**ETHNIC GROUPS**	303	30	12	160	42	12	47	X^2^ = 9.2824, df = 5, *P* = 0.09832
Indigenous African	171(56)	22(76)	7(58)	83(47)	21(50)	10(83)	28(60)	*A vs C: P* = 0.0442*
Other Ethnic groups	132 (44)	8(24)	5(42)	77(53)	21(50)	2(17)	19(40)	*E vs C: P* = 0.0393*
**AJCC TNM STAGING**	178	9	8	76	37	7	41	X^2^ = 7.6681, df = 5, *P* = 0.1755
I-II	77(43)	1(11)	4(50)	38(50)	12(32)	4(57)	18(44)	
III-IV	101(57)	8(89)	4(50)	38(50)	25(68)	3(43)	23(56)	*A vs C: P* = 0.0348*
**CROHN’S LIKE INFLAMMATORY RESPONSE**	172	9	8	73	35	7	40	X^2^ = 11.185, df = 5,*P* = 0.04782*
Mild-moderate	49(28)	6(67)	1(12)	19(26)	10(29)	4(57)	9(23)	*A vs C: P* = 0.0205*
Absent	123(72)	3(33)	7(88)	54(74)	25(71)	3(43)	31(77)	
**TUMOR INFILTRATING LYMPHOCYTES**	178	15	8	73	35	7	40	X^2^ = 12.898, df = 5, *P* = 0.02435* *D vs C: P* = 0.0497*
Mild-moderate	68(38)	5(33)	2(25)	19(26)	16(46)	5(71)	21(52)	*E vs C: P* = 0.0231*
Absent	110(62)	10(67)	6(75)	54(74)	19(54)	2(29)	19(48)	*F vs C: P* = 0.0072**

Levels of statistical significance indicated by an asterix *. *P*-values <0.05 indicated with (*), and *P*-values <0.01 with (**)

## Results

### Patient demographics

LCC revealed a higher frequency in males in IA than in OEG patients (64% vs 47%, respectively; *P* = 0.0111) (Table 1). The IA patients were younger in comparison to the OEG patients (median age: 54 vs 62 years, respectively; *P* < 0.0001).

### Pathological characterization

Signet ring cell carcinomas (SRCC) were more frequently found in IA (14/213; 7%) versus OEG patients (2/147; 1%) (*P* = 0.0221). When further stratified by site, SRCC was only associated with LCC in IA patients, as compared to OEG patients (*P* = 0.0257).

### MSI-L, MSS and MSI-H molecular subtypes

Ethnicity was linked to mononucleotide instability markers, with BAT25 and BAT26 markers being more frequently unstable in IA patients (28/29; 97%) within the MSI-L subgroup. NR21, NR24 and N27 instability was commonly demonstrated in OEG patients (7/13; 54%) (*P* = 0.0053). An increased rate of MSI-L vs MSS CRC (21% vs 9%; *P* = 0.0442) and MSI-H vs MSS CRC (11% vs 2.5%; *P* = 0.0393) was found in the left colon, particularly in IA compared to OEG patients (Table 2). In addition, the MSI-L subtype was associated with more advanced disease stage (III-IV) (8/9; 89%) when compared to MSS CRC (38/76; 50%) (*P* = 0.0348). CLR was associated with MSI-L LCC (6/9; 67%), when compared to subtypes MSI-L RCC (1/8; 12%) (*P* = 0.0498), and MSS LCC (19/73; 26%) (*P* = 0.0205). TIL was associated with subtypes MSI-H LCC (*P* = 0.0231), MSI-H RCC (*P* = 0.0072) and MSS-RCC (*P* = 0.0497).

## Discussion

CRC has been shown to have unique clinicopathological features associated with tumour site and different molecular subtypes (CMS 1–4) [11]. CRC molecular subtypes and age of onset have also been described to vary considerably among geographically distinct ethnic groups. Within this cohort, (40%) of IA patients was shown to be younger (< 50 years) compared to OEGs with pMMR CRC. Patients of OEGs in this cohort displayed RCC with poor prognostic factors compared to LCC. Increased frequencies of HG tumours (11% vs 4%) advanced staged tumours (73% vs 49%), perineural invasion (22% vs 15%), mucinous and signet ring morphology (19% vs 4%) were seen in RCC. While the median age of onset was similar for LCC vs RCC (61 vs 62), more males however were diagnosed with RCC (59%) than LCC (47%).

Within the IA patient group, both left and right colon cancers showed similar frequencies for poor prognostic factors. Higher frequencies for HG tumours (11%), advanced tumour stage (62–64%), perineural invasion (24–26%), SRCC (6%), younger age onset (median age 54–55), with more males presenting with LCC compared to RCC (64% vs 54%).

When comparing ethnic groups and right versus left-sided CRC, significant differences were observed for IA patients with LCC. The IA population group showed a propensity to occur in males, within the sigmoid colon, to present with a SRCC histological pattern, and an MSI-L status. Notably, SRCC is recognized as a rare histological subtype (1%) of CRC and is associated with young adults in other geographical locations [35]. Previous studies have shown SRCC to have a RCC dominance. However, more recently, SRCC has been reported to have an even site distribution within the colon, with a slight male predominance [36]. Moreover, the SRCC histological subtype is known to have an adverse prognostic significance independent of tumour stage and molecular subtype [37, 38]. Poor tumour grade and advanced TNM staging are usually associated with worse survival outcomes [39, 40]. In this study, these features were found to have borderline significance in IA patients with LCC compared to OEGs, with slight increases in frequencies of HG (11% vs 4%, respectively; *P* = 0.0930) and advanced disease stage (62% vs 49%, respectively; *P* = 0.0922).

When assessing PCR confirmed cases only to accurately categorize MSI-L versus MSS and MSI-H CRC in right versus left CRC, significant associations were seen for MSI-L LCC with advanced disease stage and the IA ethnic group (Table 2). This data was perceived to be similar to the findings of Devaraj 2010, linking elevated microsatellite alterations at selected tetranucleotide repeats (EMAST) with advanced disease stage, rectal cancers and peri-tumoral infiltration in patients of African American (AA) descent [41, 42]. Even though MSI-L status was not available for the data in the study of Devaraj et al. 2010, previously published data closely linked MSI-L tumours to EMAST and has been associated with a poorer prognosis [20, 22, 43]. The most frequent unstable markers in tumours from IA patients in our cohort were BAT25 (15/29; 52%) and BAT26 (13/29; 45%). MSI-L LCC tumours showed a tendency to be of a more advanced disease stage (AJCC TNM stage: III-IV) compared to MSS LCC (89% vs 50%, respectively; *P* = 0.0348).

Previous studies have shown these markers to be polymorphic within the African population, linked to the theory that older population groups show increased genetic variation [31–33]. Within this cohort, 17 CRC patients had shown instability in one or both markers (BAT25 and/or 26) in tumour and matched normal tissue, with a proficient MMR protein expression profile. These samples were assumed to be due to increased genetic polymorphisms. What was interesting and noteworthy to mention, was all these patients were exclusively of IA descent, with no differences observed in allele deletion sizes (5-15 bp deletion) between germline and somatic instability, and the majority were LCC (15/17; 88%) with advanced disease stage at diagnosis (7/10; 70%). Based on these findings, limiting as it is in size, BAT25/26 instability (whether of polymorphic/germline or somatic variation) was associated with advanced disease stage in proficient MMR LCC patients of IA descent. Even though survival data was not available for this study, literature has shown poor clinical prognosis and overall survival associated with MSI-L CRC, particularly for advanced disease stage CRC [21–23, 44]. The somatic MSI-L LCC group was associated with CLR when compared to MSI-L RCC (67% vs 12%; *P* = 0.0498) and MSS LCC (67% vs 26%; *P* = 0.0205). Polymorphic/germline MSI-L tumours within 2 markers (11/17; 65%) however displayed no CLR. Literature has indicated tumours with CLR to have a better prognosis compared to stage-matched tumours without [45–47]. This raises a plausible argument that germline MSI-L tumours could have a worse prognosis compared to somatic MSI-L tumours, due to the lack of the host’s immune response to the cancer.

BAT26 marker (26(A) repeats) is located in intron 5 of the MSH2 gene on chromosome 2p21. This marker is situated immediately downstream of exon 5, which is susceptible to large intragenic deletions and accounts for nearly a third of dMSH2 mutations [48–50]. Studies by Pastrello et al. 2006 and Jaskowski et al. 2007 indicated that instability in Bat26 was associated with overall instability of dMSH2 tumours. Confirmatory IHC to determine dMMR protein expression is therefore important, however exceptions of cases with mutations in intronic nucleotides close to splice sites could result in expressed non-functional proteins, such as (*MSH2* c.913G > A p.Ala305Thr) which has been reported with proficient MMR activity and a MSI-L genotype [50]. This variant however had no aberrant splicing and normal subcellular localization and interaction with MSH6 was shown [50, 51].

BAT25 (25(T) repeats) is situated within intron 16 of the c-kit proto-oncogene on chromosome 4q12. cKit (CD117) the receptor for Stem cell factor (SCF) involved in haemopoiesis has more recently shown to be involved in lymphopoiesis. The CD117 receptor has shown to be expressed on mature CD8^+^ T cells following initial activation, suppressing differentiation and increasing its response to apoptosis. CD117 expressed CD8^+^ T cells could therefore play a role in CD117-blockade, an important mechanism in tumour immune evasion. BAT25 instability in the CD117 gene could potentially play a role in immune evasion in MSI-L CRC, and additional studies are required to determine CLR and its association with MSI-L LCC.

A study by Carethers et al. 2014 showed an increased incidence of MSI-H CRC in AA patients, however had poorer prognosis and higher mortality rates compared to their Caucasian counterparts [52]. This was thought to be due to AA patients showing a lower infiltration of CD8^+^ T cells compared to Caucasian patients, suggesting an altered immune function in AA patients. It has been well established that the increased tumour-infiltrating CD4^+^ and CD8^+^ T cells in patients with MSI-H CRC (due to the increased mutator phenotype of the tumour stimulating the host immune response) significantly improved patient outcomes when treated with immunotherapy when compared to MSS CRC with decreased immune response [53, 54]. The study by Carethers et al. 2014 illustrate despite having the same disease subtype and stage, ethnicity can be a negative prognostic factor in CRC disease.

A frequency of 17% MSI-L CRC in SA CRC patients has been reported in our previous study [29]. Further evaluation in this study has demonstrated MSI-L LCC to occur predominantly in IA patients, and associated with advanced disease stage, with considerable number of germline/polymorphic MSI-L LCC also presenting at an advance stage compared to MSI-L RCC and MSS LCC.

Based on these findings, universal MSI PCR screening is recommended as a first-line screening method for all newly diagnosed CRC patients, to not only identify MSI-H CRC, but also increase the detection rate of MSI-L CRC. Local ethnic polymorphisms however have to be taken into account when implementing diagnostic marker panels in certain geographical settings. If instability is required in 30% of markers used in a panel for a diagnosis of MSI, it is important to confirm markers included are non-polymorphic in the local population.

CRC is a heterogeneous disease and more studies are required to unravel the complexity associated with it by investigating different ethnic groups in the context of site. This study has shown that ethnicity and tumour site play an important role in the prognostication of tumours and should be taken into consideration for effective treatment planning, especially in geographical regions with diverse population groups such as South Africa. Limitations of this study include selection bias, as only samples with an MSI status were included. This resulted in smaller sample sizes for the analysis of certain features such as polyps, TILs, TNM staging, MSI-L and BAT25/26 instability status. The lack of universal screening for MSI within the study institution, as well as the inclusion of biopsy samples in addition to resections, have contributed to providing limited information. Confirmation of MSI-L status in biopsies was not possible, increasing the likelihood of a small percentage of false positive MSI-L samples with normal variation.

A study by Ozaki et al. found that MSI-L colon tissue occurred in a few but not all intestinal crypts, and both in malignant and normal tissues. The presence of MSI-L in non-neoplastic mucosa could indicate a primary step in tumorigenesis and could potentially be used as an early diagnostic and prognostic marker in CRC [55].

Additional AA patient studies have illustrated increased frequencies of MSI-L/EMAST markers in rectal cancers most likely due to somatic inactivation of an alternative MMR gene (MSH3) [21, 44, 56, 57]. Dysfunctional MSH3 has shown to lead to MSI, appearing to be inflammation-related within the tumour microenvironment [58]. Regular intake of anti-inflammatory drugs such as aspirin and non-steroidal anti-inflammatory drugs (NSAID) has been reported to prevent the development of colorectal adenomas, tumour growth and progression, as well recurrence and metastasis after curative surgery, prolonging colorectal cancer patient survival [59–61]. Anti-inflammatories could therefore possibly have a positive effect not only in MSI-H CRC, but also in this subgroup of MSI-L LCC patients. In addition, due to the presence of CLR, MSI-L LCC could potentially be an eligible subgroup for immunotherapeutic strategies in metastatic disease and further studies are recommended.

## Conclusion

This SA CRC study indicated that in considering categorization of CRC according to anatomical site, microsatellite instability status and ethnicity, unique clinicopathological features were identified. In particular, IA CRC patients with LCC are more likely to be male, have an MSI-L subtype, show BAT25/BAT26 marker instability and have advanced disease stage. This study suggests that BAT25 and BAT26 instability are negative prognostic markers in African CRC patients, and larger confirmatory studies are recommended. Further exploratory studies of MSH3, EMAST, KRAS and immune cell infiltration in the tumour microenvironment are indicated in SA CRC patients. This will assist in establishing molecular profiles to accurately improve diagnostic, prognostic and personalized predictive markers for the effective management of early onset CRC.

## Data Availability

The dataset generated and analysed during the current study are available from the corresponding author on reasonable request.

## References

[1] Iacopetta B (2002). Are there two sides to colorectal cancer?. Int J Cancer.

[2] Mik M, Berut M, Dziki L, Trzcinski R, Dziki A (2017). Right-and left-sided colon cancer-clinical and pathological differences of the disease entity in one organ. Arch Med Sci.

[3] Lim DR, Kuk JK, Kim T, Shin EJ (2017). Comparison of oncological outcomes of right-sided colon cancer versus left-sided colon cancer after curative resection. Med (United States).

[4] Benedix F (2010). Comparison of 17,641 patients with right- and left-sided colon cancer: differences in epidemiology, perioperative course, histology, and survival. Dis Colon Rectum.

[5] Holch JW, Ricard I, Stintzing S, Modest DP, Heinemann V (2017). The relevance of primary tumour location in patients with metastatic colorectal cancer: A meta-analysis of first-line clinical trials. Eur J Cancer.

[6] Baran B (2018). Difference Between Left-Sided and Right-Sided Colorectal Cancer: A Focused Review of Literature. Gastroenterol Res.

[7] Medhanie GA (2017). Cancer incidence profile in sub-Saharan African-born blacks in the United States: Similarities and differences with US-born non-Hispanic blacks. Cancer.

[8] Walker ARP, Segal I (2002). Colorectal cancer in an African city population in transition. Eur J Cancer Prev.

[9] Katsidzira L (2017). The shifting epidemiology of colorectal cancer in sub-Saharan Africa. Lancet Gastroenterol Hepatol.

[10] Center MM, Jemal A, Smith RA, Ward E (2009). Worldwide variations in colorectal cancer. CA Cancer J Clin.

[11] Guinney J (2015). The consensus molecular subtypes of colorectal cancer. Nat Med.

[12] Kim SY, Kim T Il. Serrated neoplasia pathway as an alternative route of colorectal cancer carcinogenesis. Intest Res. 2018;16:358–65. 10.5217/ir.2018.16.3.358 PMC6077295 30090034

[13] Stintzing S, Tejpar S, Gibbs P, Thiebach L, Lenz HJ (2017). Understanding the role of primary tumour localisation in colorectal cancer treatment and outcomes. Eur J Cancer.

[14] Samowitz WS (2005). Evaluation of a large, population-based sample supports a CpG island methylator phenotype in colon cancer. Gastroenterology.

[15] Muller M, Ibrahim A, Arends M (2016). Molecular pathological classification of colorectal cancer. Virchows Arch.

[16] Ogino S (2009). CpG island methylator phenotype, microsatellite instability, BRAF mutation and clinical outcome in colon cancer. Gut.

[17] Hile SE, Shabashev S, Eckert KA (2013). Tumor-specific microsatellite instability: Do distinct mechanisms underlie the MSI-L and EMAST phenotypes? Mutat. Res - Fundam Mol Mech Mutagen.

[18] Mori Y (2003). The impact of microsatellite instability on the molecular phenotype of colorectal tumors. Cancer Res.

[19] Pawlik TM, Raut CP, Rodriguez-bigas MA (2004). Colorectal carcinogenesis : MSI-H versus MSI-L.

[20] TorshiziEsfahani A, Seyedna SY, NazemalhosseiniMojarad E, Majd A, AsadzadehAghdaei H (2018). MSI-L/EMAST is a predictive biomarker for metastasis in colorectal cancer patients. J Cell Physiol.

[21] Lee SY (2015). Low-level microsatellite instability as a potential prognostic factor in sporadic colorectal cancer. Med (United States).

[22] Mojarad EN (2016). Low Level of Microsatellite Instability Correlates with Poor Clinical Prognosis in Stage II Colorectal Cancer Patients. J Oncol.

[23] Wright CM (2005). Low level microsatellite instability may be associated with reduced cancer specific survival in sporadic stage C colorectal carcinoma. Gut.

[24] Jass JR (2007). Classification of colorectal cancer based on correlation of clinical, morphological and molecular features. Histopathology.

[25] Cronjé L, Paterson AC, Becker PJ (2009). Colorectal cancer in South Africa: A heritable cause suspected in many young black patients. South African Med J.

[26] Prodehl L, Bebington B, Fabian J, Singh E, Ruff P (2017). COLORECTAL CANCER IN A SOUTH AFRICA URBAN SETTING - A PRELIMINARY ANALYSIS. S Afr J Surg.

[27] Bebington B (2018). Design and methodology of a study on colorectal cancer in Johannesburg. South Africa JGH Open.

[28] McCabe M, Perner Y, Magobo R, Mirza S, Penny C (2020). Descriptive epidemiological study of South African colorectal cancer patients at a Johannesburg Hospital Academic institution. JGH Open.

[29] McCabe M (2019). Microsatellite Instability assessment in Black South African Colorectal Cancer patients reveal an increased incidence of suspected Lynch syndrome. Sci Rep.

[30] Haghighi MM (2010). Frequent MSI mononucleotide markers for diagnosis of hereditary nonpolyposis colorectal cancer. Asian Pac J Cancer Prev.

[31] Pyatt R (1999). Implications for Microsatellite Instability Testing. Cancer Res.

[32] Suraweera N (2002). Evaluation of tumor microsatellite instability using five quasimonomorphic mononucleotide repeats and pentaplex PCR. Gastroenterology.

[33] Buhard O (2005). Multipopulation Analysis of Polymorphisms in Five Mononucleotide Repeats Used to Determine the Microsatellite Instability Status of Human Tumors. J Clin Oncol.

[34] Brennetot C (2005). Mononucleotide repeats BAT-26 and BAT-25 accurately detect MSI-H tumors and predict tumor content: Implications for population screening. Int J Cancer.

[35] Farraj FA, Sabbagh H, Aridi T, Fakhruddin N, Farhat F (2019). Signet Ring Cell Carcinoma of the Colon in Young Adults: A Case Report and Literature Review. Case Rep Oncol Med.

[36] Wei Q (2016). Clinicopathologic and molecular features of colorectal adenocarcinoma with signet-ring cell component. PLoS ONE.

[37] Hamilton S, Bosman F, Boffetta P, Ilyas M, Morreau H, Nakamura S, et al. Tumours of the colon and rectum: Chapter 8. In: Bosman FT, Carneiro F, Hruban RH, D TN, editors. WHO Classification of Tumours of the Digestive System 2010. 4th ed. IARC: Lyon 2010; 2010. p. 137–8.

[38] Hartman DJ (2013). Signet Ring Cell Colorectal Carcinoma. Am J Surg Pathol.

[39] Schneider NI, Langner C (2014). Prognostic stratification of colorectal cancer patients: Current perspectives. Cancer Manag Res.

[40] Fleming M, Ravula S, Tatishchev SF, Wang HL (2012). Colorectal carcinoma: Pathologic aspects. J Gastrointest Oncol.

[41] Devaraj B (2010). Relationship of EMAST and Microsatellite Instability Among Patients with Rectal Cancer. J Gastrointest Surg.

[42] Lee SY (2012). Microsatellite instability, EMAST, and morphology associations with T cell infiltration in colorectal neoplasia. Dig Dis Sci.

[43] Garcia M (2012). Association between recurrent metastasis from stage II and III primary colorectal tumors and moderate microsatellite instability. Gastroenterology.

[44] Kohonen-Corish MRJ (2005). Low microsatellite instability is associated with poor prognosis in stage C colon cancer. J Clin Oncol.

[45] Buckowitz A (2005). Microsatellite instability in colorectal cancer is associated with local lymphocyte infiltration and low frequency of distant metastases. Br J Cancer.

[46] Rozek LS (2016). Tumor-Infiltrating lymphocytes, Crohn’s-like lymphoid reaction, and survival from colorectal cancer. J Natl Cancer Inst.

[47] Posch F (2018). Maturation of tertiary lymphoid structures and recurrence of stage II and III colorectal cancer. Oncoimmunology..

[48] Pastrello C (2006). Stability of BAT26 in tumours of hereditary nonpolyposis colorectal cancer patients with MSH2 intragenic deletion. Eur J Hum Genet.

[49] Jaskowski L (2007). Stability of BAT26 in Lynch syndrome colorectal tumours [1]. Eur J Hum Genet.

[50] Arnold S (2009). Classifying MLH1 and MSH2 variants using bioinformatic prediction, splicing assays, segregation and tumor characteristics Sven. Hum Mutat.

[51] Thompson BA, Martins A, Spurdle AB (2015). A review of mismatch repair gene transcripts: Issues for interpretation of mRNA splicing assays. Clin Genet.

[52] Carethers JM (2014). Influence of race on microsatellite instability and CD8+ T cell infiltration in colon cancer. PLoS ONE.

[53] Wang J, Liu J, Tian F, Zhan Y, Kong D (2019). Cyclin-dependent kinase 9 expression and its association with CD8+ T cell infiltration in microsatellite-stable colorectal cancer. Oncol Lett.

[54] Frumento G, Zuo J, Verma K, Croft W, Ramagiri P, Chen FE, et al. CD117 (c-Kit) is expressed during CD8+ T cell priming and stratifies sensitivity to apoptosis according to strength of TCR engagement. Front Immunol. 2019;10:468. 10.3389/fimmu.2019.00468 PMC6428734 30930902

[55] Ozaki K, Nagasaka T, Notohara K, Kambara T, Takeda M, Sasamoto H, et al. Heterogeneous microsatellite instability observed within epithelium of ulcerative colitis. Int J Cancer. 2006;119:2513–9. 10.1002/ijc.22095 16929496

[56] Ashktorab H (2015). Identification of novel mutations by exome sequencing in African American colorectal cancer patients. Cancer.

[57] Carethers JM, Koi M, Tseng-Rogenski SS (2015). EMAST is a form of microsatellite instability that is initiated by inflammation and modulates colorectal cancer progression. Genes (Basel).

[58] Koi M, Tseng-Rogenski SS, Carethers JM (2018). Inflammation-associated microsatellite alterations: Mechanisms and significance in the prognosis of patients with colorectal cancer. World J Gastrointest Oncol.

[59] Cole BF (2009). Aspirin for the chemoprevention of colorectal adenomas: Meta-analysis of the randomized trials. J Natl Cancer Inst.

[60] Arber N (2006). Celecoxib for the prevention of colorectal adenomatous polyps. N Engl J Med.

[61] Hamada T (2017). Aspirin use and colorectal cancer survival according to tumor CD274 (programmed cell death 1 ligand 1) expression status. J Clin Oncol.

